# The role of biochar in enhancing soil health & interactions with rhizosphere properties and enzyme activities in organic fertilizer substitution

**DOI:** 10.3389/fpls.2025.1595208

**Published:** 2025-06-13

**Authors:** Aamir Ali, Nida Jabeen, Zaid Chachar, Sadaruddin Chachar, Shoaib Ahmed, Nazir Ahmed, Azhar Ali Laghari, Zulfiqar Ali Sahito, Rasulov Farruhbek, Zhenping Yang

**Affiliations:** ^1^ College of Agriculture, Shanxi Agricultural University, Jinzhong, China; ^2^ Department of Pharmaceutical Sciences, Andijan State Medical Institute, Andijan, Uzbekistan; ^3^ School of Communications and Information Engineering, Chongqing University of Posts and Telecommunications, Chongqing, China; ^4^ College of Agriculture and Biology, Zhongkai University of Agriculture and Engineering, Guangzhou, China; ^5^ College of Horticulture and Landscape Architecture, Zhongkai University of Agriculture and Engineering, Guangzhou, China; ^6^ Department of Agronomy, Sindh Agriculture University, Tandojam, Pakistan; ^7^ Department of Agronomy, Faculty of Crop Production, Sindh Agriculture University, Tandojam, Pakistan; ^8^ College of Resources and Environment, Shanxi Agricultural University, Shanxi, Jinzhong, China; ^9^ Industrial Crops Research Institute, Yunnan Academy of Agricultural Sciences (YAAS), Kunming, Yunnan, China

**Keywords:** biochar, organic fertilizers, nutrient cycling, carbon sequestration, sustainable agriculture

## Abstract

Modern agriculture faces a dual challenge: sustainable crop production and reducing the environmental impacts of excessive chemical fertilizers use, which leads to soil degradation, nutrient leaching and declining microbial diversity. Addressing these issues, biochar, a carbon-rich by product of pyrolysis, has emerged as a promising soil amendment due to its ability to enhance soil health, support nutrient cycling, and contribute to climate mitigation. However, its interactive effects with rhizosphere dynamics and soil enzymatic process, particularly when used with organic fertilizers, remain insufficiently explored. This review compiles current knowledge on the short-term and long-term impacts of biochar, particularly in combination with organic fertilizers, on rhizosphere properties, enzyme activities, and nutrient dynamics. In the short term, biochar improves soil structure, water retention, and microbial activity, while reducing nutrient leaching and increasing enzymatic functions. Over the long term, it facilitates carbon sequestration, stabilizes soil organic matter (SOM), and ensures nutrient availability, thereby promoting sustainable crop production. The synergistic application of biochar with organic amendments, such as compost and crop residues, further enhances soil fertility and ecosystem services. Despite its numerous benefits, the adoption of biochar on a larger scale is hindered by challenges related to cost-effectiveness, production consistency, and logistical constraints in diverse agricultural systems. Addressing knowledge gaps related to optimal feedstock selection, pyrolysis conditions, and application rates is essential for maximizing biochar’s potential. By integrating biochar into sustainable agricultural practices, farmers can enhance soil productivity, reduce environmental impacts, and contribute to climate change mitigation. A strategic and evidence-based implementation of biochar technologies holds promise for achieving long-term sustainability and food security goals.

## 1 Introduction

Sustainable agriculture is increasingly critical in achieving food security, environmental protection, and long-term ecosystem health. A key element in this transformation is reducing dependency on synthetic chemical fertilizers, a practice that has been central to modern agriculture (Muhie, 2022). However, the over-reliance on these fertilizers has led to serious adverse outcomes. Although they provide essential nutrients, their widespread use has contributed to soil degradation, water contamination from nutrient runoff, and a decline in soil biodiversity. Moreover, the energy demands for producing and transporting chemical fertilizers add significantly to greenhouse gas emissions, further exacerbating climate change (Muhie, 2022).

The over-reliance on chemical fertilizers has led to several adverse environmental and economic consequences. While chemical fertilizers provide essential nutrients to crops, their widespread use has resulted in soil degradation, water contamination through nutrient runoff, and the depletion of soil biodiversity (Ahmed et al., 2024). Additionally, the energy-intensive production and transportation of chemical fertilizers contribute significantly to greenhouse gas emissions, exacerbating climate change. These issues highlight the urgent need for sustainable alternatives that can maintain or enhance agricultural productivity while minimizing environmental harm (Pahalvi et al., 2021).

One promising solution to this challenge is the use of organic amendments, such as bio-organic fertilizers (BOFs), which combine the benefits of organic materials and bio-based products. These amendments not only help reduce the reliance on synthetic fertilizers but also improve soil health, enhance microbial activity, and promote more efficient nutrient cycling. Among these, biochar, a stable form of carbon produced through the pyrolysis of organic materials, has emerged as a powerful soil amendment with significant potential to enhance soil fertility and improve agricultural sustainability (Bamdad et al., 2022). This paper reviews the role of biochar in sustainable agriculture, focusing on its interactions with rhizosphere properties, soil enzymes, and its potential to reduce the dependency on chemical fertilizers. Through this review, we aim to highlight the importance of integrating biochar into agricultural systems to foster long-term sustainability and reduce environmental impacts (Karim et al., 2022).

Historically, biochar has roots in traditional agricultural practices. One of the most well-known examples is the Terra Preta soils of the Amazon Basin, where indigenous peoples enriched the soil with charred biomass, creating highly fertile and productive lands (Manikandan et al., 2023). These practices not only improved soil fertility but also provided a lasting carbon sink, illustrating the long-term benefits of biochar for both agriculture and the environment.

Biochar is a highly porous, carbon-rich material produced through the thermal decomposition of organic biomass in a low-oxygen environment, a process known as pyrolysis (Irshad et al., 2023). This process stabilizes the carbon content of the organic material, creating a durable product that can remain in the soil for centuries. Biochar enhances soil fertility by improving soil structure, nutrient retention and microbial interactions, making it a valuable tool in sustainable agriculture (Samaraweera et al., 2023). The production of biochar involves heating organic feedstocks such as agricultural residues, forestry by-products, or municipal waste at temperatures typically ranging from 300°C to 700°C in an oxygen-limited environment. The resulting product retains a significant fraction of the carbon from the original biomass while releasing volatile gases and bio-oil as by-products. These by-products can serve as renewable energy sources, contributing to the circular economy and reducing overall carbon emissions (Patel and Panwar, 2023).

Today, biochar is gaining renewed interest as a sustainable amendment for modern agriculture, offering a range of environmental benefits such as carbon sequestration, nutrient cycling, and mitigation of greenhouse gas emissions. As an integral part of sustainable soil management practices, biochar holds the potential to address some of the most pressing challenges in agricultural production, including soil degradation, nutrient loss, and climate change (Khare et al., 2021).

The intensification of agricultural practices to meet the growing global food demand has led to significant environmental challenges, including soil degradation, nutrient imbalances, and greenhouse gas emissions (Kopittke et al., 2019). These challenges are further exacerbated by the overuse of chemical fertilizers, which, while effective in providing essential nutrients, often result in long-term soil health decline and environmental contamination. Consequently, there is a pressing need for sustainable soil management strategies that balance productivity with environmental stewardship. Biochar has emerged as a promising solution to address these challenges. Its unique physical and chemical properties make it a powerful soil amendment capable of improving soil structure, water retention, and nutrient availability. Furthermore, biochar’s high stability allows it to sequester carbon in the soil for extended periods, contributing to climate change mitigation. Beyond these benefits, biochar has demonstrated the potential to enhance soil enzymatic activities, which play a critical role in nutrient cycling and organic matter decomposition (Al Masud et al., 2023). These enzymes facilitate the breakdown of organic compounds, releasing nutrients into forms that plants can readily absorb, thereby improving overall soil fertility.

When used in combination with organic fertilizers, such as bio-organic fertilizers (BOFs) or organic manure, biochar further amplifies these benefits. Organic fertilizers provide a source of nutrients and organic matter, while biochar acts as a stabilizing agent, retaining nutrients and creating a favorable environment for microbial activity (Hu et al., 2024). This synergy not only improves nutrient use efficiency but also promotes long-term soil health by supporting enzymatic functions and reducing nutrient leaching. Given the increasing interest in biochar as a sustainable agricultural amendment, this review aims to explore its role in enhancing soil properties and enzyme activities, particularly in conjunction with organic fertilizers. By synthesizing current knowledge, this paper seeks to provide insights into the mechanisms, benefits, and potential applications of biochar in sustainable agriculture, offering a pathway toward reduced dependency on chemical fertilizers and improved environmental outcomes.

While numerous studies have explored the benefits of biochar in soil fertility and carbon sequestration, limited reviews have systematically examined its synergistic interactions with organic fertilizers in modifying rhizosphere-specific properties and enzyme activities. This review uniquely consolidates short- and long-term effects of biochar–organic fertilizer integration, with a focus on nutrient cycling, microbial functions, and sustainable nutrient management. By emphasizing rhizosphere-specific responses, this work offers novel insights into optimizing soil amendment strategies for sustainable agriculture under varying agroecological conditions.

This study is based on the hypothesis that integrating biochar with organic fertilizers significantly enhances rhizosphere properties and enzyme activities, contributing to improved soil fertility, nutrient cycling, and sustainable crop production.

## 2 Rhizosphere soil properties and their importance in plant growth

The rhizosphere is the narrow zone of soil around the plant roots. This unique microenvironment is characterized by complex physical, chemical, and biological interactions between plant roots, soil particles, and soil microorganisms. The rhizosphere is often referred to as the “hotspot” of soil activity due to the intense biochemical processes that occur within this zone (The Rhizosphere: Contributions of the Soil – Root Interface to Sustainable Soil Systems, 2020). A key factor shaping rhizosphere activity is root exudation. In the rhizosphere, plant roots exude organic compounds such as sugars, amino acids, and secondary metabolites, collectively known as root exudates (Vives-Peris et al., 2020). These exudates serve as a source of energy and nutrients for soil microorganisms, fostering a diverse and dynamic microbial community. This interaction between roots and microbes creates a symbiotic relationship where microbes enhance nutrient availability, suppress pathogens, and improve soil structure, while plants provide a steady supply of carbon compounds (Ahmed et al., 2023).

The rhizosphere plays a pivotal role in plant growth and development by regulating nutrient acquisition, water uptake, and root architecture. It also acts as a critical interface between plants and their soil environment, influencing the overall health and productivity of crops. Understanding the processes within the rhizosphere is essential for developing strategies to improve soil fertility, enhance plant resilience to stress, and promote sustainable agricultural practices (Solomon et al., 2024). The rhizosphere is not only a dynamic zone where plant roots interact with soil microorganisms but also a region where several key soil properties influence plant growth and nutrient uptake (Hussain et al., 2024). These properties soil texture, structure, and porosity; pH; and water retention and drainage are critical for maintaining healthy soil environments and promoting efficient plant development. Below, we explore how each of these properties impacts the rhizosphere and overall plant health.

Soil texture refers to the relative proportions of sand, silt, and clay particles in the soil, and it plays a significant role in determining the soil’s ability to retain and transmit water and nutrients. In the rhizosphere, soil texture influences the movement of water, oxygen, and nutrients to and from plant roots. For example, sandy soils, with their larger particles, tend to have good drainage but lower nutrient retention, while clay soils, with smaller particles, retain water and nutrients better but may suffer from poor drainage and aeration (Guo et al., 2022). Soil structure referring to the arrangement of soil particles into aggregates affects root penetration, microbial activity, and water infiltration. Well-structured soils, with a balanced proportion of large and small aggregates, facilitate deeper root growth and allow for more efficient exchange of gases and nutrients between roots and soil microorganisms (Basset et al., 2023). Soil porosity, the volume of pore spaces between soil particles, is another important factor. Adequate porosity ensures that the rhizosphere has enough air for root respiration and microbial activity, while also allowing water to percolate through the soil, preventing waterlogging (Morya et al., 2023). Soil pH directly affects nutrient availability in the rhizosphere. Most plants thrive in soils with a pH between 5.5 and 7.0, where nutrients are most readily available for uptake. At low pH (acidic soils), certain essential nutrients such as phosphorus, calcium, and magnesium become less available, while toxic elements like aluminum can increase, potentially harming plant roots. On the other hand, alkaline soils (high pH) can result in nutrient imbalances, such as deficiencies in iron, manganese, and zinc, which are essential for plant growth (Dewangan et al., 2023).

Biochar, when applied to soil, can help regulate pH by acting as a buffering agent. This can be particularly beneficial in acidic soils, as biochar has been shown to increase soil pH and improve the availability of key nutrients, making it a valuable amendment for optimizing nutrient uptake in the rhizosphere (Dai et al., 2017). Water availability is a key determinant of plant growth and development, and it is strongly influenced by the water retention and drainage capacity of the soil. In the rhizosphere, water retention is essential for maintaining a constant supply of moisture for root absorption, particularly during periods of drought. However, poor drainage can lead to waterlogged conditions that limit root oxygen supply and promote the growth of pathogenic microorganisms (Jaramillo-Quiceno et al., 2024). Soil amendments like biochar can improve water retention in sandy soils and enhance drainage in clayey soils, thereby promoting a healthier and more balanced rhizosphere environment. Biochar’s high porosity enables it to retain moisture while also facilitating aeration, which benefits both plant roots and soil microbes.

**Figure 1:**
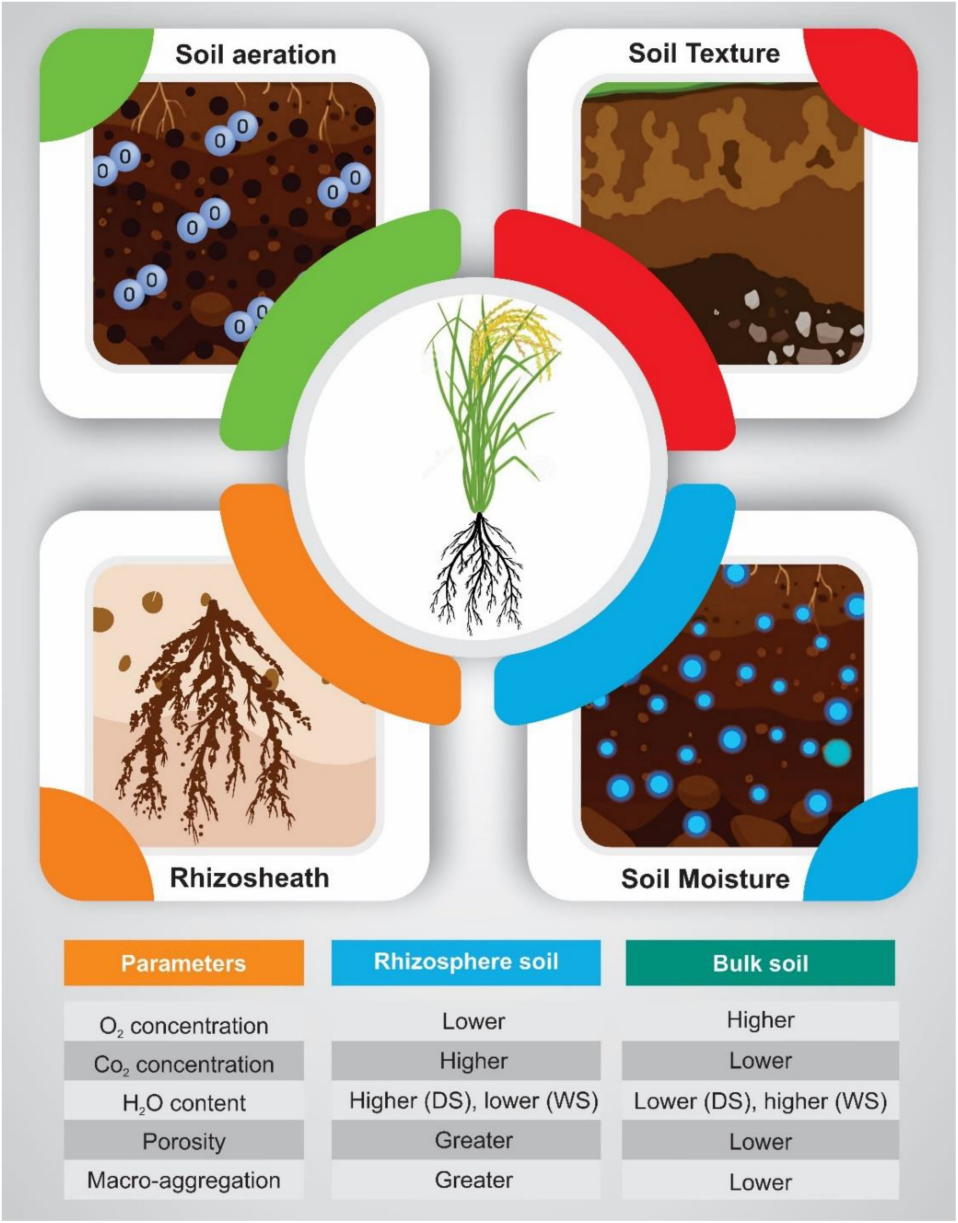
Comparison of soil properties between rhizosphere and bulk soil. The schematic illustrates key differences in soil aeration, moisture, and texture around plant roots. Insets highlight variations in oxygen and carbon dioxide concentrations, soil porosity, and macro-aggregation between rhizosphere and bulk soil. The accompanying table summarizes quantitative differences, showing that rhizosphere soil has lower oxygen concentration, higher carbon dioxide concentration, increased porosity, and greater macro-aggregation than bulk soil. Soil moisture content fluctuates based on environmental conditions, with rhizosphere soil retaining more moisture during dry periods but less during wet conditions.

The comparison between rhizosphere soil and bulk soil revealed significant differences in key soil properties. Oxygen concentration was lower in rhizosphere soil compared to bulk soil, whereas carbon dioxide concentration was higher, indicating increased root respiration and microbial activity near the root zone. Soil moisture content varied with environmental conditions, being higher in the rhizosphere during dry spells (DS) but lower than bulk soil in wet conditions (WS). Additionally, rhizosphere soil exhibited greater porosity and macro-aggregation, suggesting improved soil structure and aeration around the roots. These characteristics, along with the formation of the rhizosheath, contribute to an enhanced root-soil interface, which likely facilitates nutrient and water uptake (**Figure 1**).

### 2.1 The role of soil amendments in modifying rhizosphere properties

Soil amendments such as (BOFs)and organic manure play a crucial role in enhancing rhizosphere properties, improving soil fertility, and supporting plant growth (Kang et al., 2022). These organic amendments not only supply essential nutrients to plants but also positively influence the physical, chemical, and biological characteristics of the rhizosphere. Below, we discuss how these amendments contribute to modifying key rhizosphere properties.

(BOFs)and organic manure are rich sources of organic matter, which is a critical component of healthy soils. When applied to the rhizosphere, these amendments increase the (SOM) content, improving soil structure, aeration, and water retention. Organic matter also serves as a substrate for soil microorganisms, boosting microbial activity and supporting nutrient cycling processes (Singh et al., 2020a). Organic amendments release nutrients gradually, providing a sustained supply of essential elements such as nitrogen, phosphorus, and potassium. This slow-release mechanism improves nutrient availability in the rhizosphere while reducing the risk of nutrient leaching and environmental contamination. For example, nitrogen in organic manure is converted into plant-available forms through microbial processes, enhancing its uptake by roots (Aytenew and Bore, 2020). Organic amendments improve soil aggregation, which enhances the structural stability of the rhizosphere. Better soil structure facilitates root penetration, increases porosity, and improves the movement of air and water within the soil. Organic manure, in particular, is effective in binding soil particles into aggregates, reducing soil compaction and creating a more favorable environment for root growth (Luo et al., 2018). In combination with biochar, these amendments can further enhance soil porosity and water-holding capacity, providing additional benefits to plants during periods of water stress (de Castro et al., 2023).

The application of organic amendments can help buffer soil pH, making it more favorable for nutrient uptake. For instance, organic manure often contains calcium and magnesium, which can neutralize soil acidity and improve the availability of nutrients such as phosphorus. (BOFs), which include microbial inoculants, may also promote pH regulation by altering microbial activity and nutrient cycling in the rhizosphere (Sazali et al., 2024). One of the most significant benefits of (BOFs)and organic manure is their ability to stimulate microbial activity in the rhizosphere (Mehata et al., 2023). These amendments provide an energy source for beneficial microbes, fostering a diverse and active microbial community. Microorganisms in the rhizosphere play essential roles in decomposing organic matter, fixing atmospheric nitrogen, solubilizing phosphorus, and suppressing plant pathogens.

(BOFs), which combine organic materials with specific microbial inoculants, can further enhance these processes by introducing beneficial microbes such as nitrogen-fixing bacteria (*Rhizobium*), phosphate-solubilizing bacteria, and mycorrhizal fungi into the rhizosphere (Shahwar et al., 2023). This symbiotic relationship between plants and microbes improves nutrient availability and uptake, leading to healthier and more productive crops. Organic amendments also influence water dynamics in the rhizosphere. By increasing (SOM), these amendments improve the soil’s ability to retain water, ensuring a steady supply of moisture to plant roots. At the same time, they enhance soil drainage, preventing waterlogging and creating a more balanced moisture regime in the rhizosphere (Ayangbenro et al., 2022).

When combined with biochar, the water-holding capacity of the soil can be further enhanced, as biochar’s porous structure allows it to store water and release it slowly to plants (Adhikari et al., 2022). This synergistic effect makes the combination of (BOFs), organic manure, and biochar particularly effective in improving water management in the rhizosphere. (BOFs) and organic manure are vital tools for modifying rhizosphere properties to promote plant health and soil fertility. Their ability to enhance (SOM), improve structure, regulate pH, stimulate microbial activity, and optimize water dynamics makes them indispensable in sustainable agricultural practices. When used in combination with biochar, these amendments offer even greater potential for improving rhizosphere properties and achieving long-term agricultural sustainability. Building upon this understanding of how organic amendments influence rhizosphere properties, the next section delves into the specific roles and mechanisms of organic fertilizers in enhancing soil fertility and microbial dynamics.

## 3 The impact of organic fertilizers on rhizosphere properties

Organic fertilizers, including organic manure and (BOFs), play a pivotal role in enhancing rhizosphere properties. By supplying essential nutrients, increasing (SOM), and improving nutrient retention, these amendments create a more favorable environment for plant growth and soil microbial activity. Below, we explore their composition, nutrient release patterns, and effects on critical soil properties such as (SOM)(SOM) content and cation exchange capacity (CEC) (Singh et al., 2020b). Organic fertilizers are composed of organic materials derived from plant and animal sources. Organic manure typically includes decomposed plant residues, animal waste, or compost, which are rich in macronutrients (nitrogen, phosphorus, potassium) and micronutrients (iron, zinc, manganese). (BOFs), in addition to organic materials, incorporate beneficial microorganisms such as nitrogen-fixing bacteria, phosphate-solubilizing bacteria, and mycorrhizal fungi, enhancing their impact on nutrient cycling and plant health (Zhao et al., 2024).

A key characteristic of organic fertilizers is their slow and steady nutrient release. Unlike chemical fertilizers, which provide an immediate nutrient boost, organic fertilizers decompose gradually, releasing nutrients over time as they are broken down by soil microbes. This sustained nutrient supply ensures that plants receive a consistent flow of essential elements while minimizing the risks of nutrient leaching and volatilization. The microbial inoculants in (BOFs)further accelerate the decomposition process and enhance nutrient availability in the rhizosphere (Venugopalan et al., 2022). One of the most significant benefits of organic fertilizers is their ability to increase (SOM) content. SOM is a key indicator of soil health and fertility, as it directly influences soil structure, water-holding capacity, and nutrient cycling (Assefa, 2019). When organic manure or (BOFs)are applied, they add carbon-rich organic material to the soil. This material serves as a food source for soil microbes, which decompose the organic matter into humus, a stable form of organic carbon that improves soil aggregation and supports long-term soil fertility (Anuradha and Singh, 2021).

In the rhizosphere, increased SOM enhances soil aeration and root penetration, creating a more conducive environment for plant roots and microorganisms. Additionally, the organic matter retains water and nutrients, reducing the need for frequent irrigation and fertilization. This improvement in SOM content contributes to the overall resilience of the soil and supports sustainable agricultural practices.

### 3.1 Influence on cation exchange capacity and nutrient retention

Cation exchange capacity (CEC) is a critical soil property that measures the soil’s ability to retain and exchange positively charged ions (cations) such as calcium (Ca²^+^), magnesium (Mg²^+^), potassium (K^+^), and ammonium (NH₄^+^). Organic fertilizers, particularly those rich in organic matter, play a crucial role in enhancing CEC in the rhizosphere (Ross and Skyllberg, 2023). As organic manure and (BOFs) decompose, they contribute humic substances that increase the soil’s CEC. This improvement allows the soil to hold more nutrients in plant-available forms, reducing nutrient leaching and improving nutrient use efficiency. The enhanced nutrient retention ensures that essential elements remain accessible to plant roots over time, even under conditions of heavy rainfall or irrigation (Prasad et al., 2017). (BOFs), with their microbial inoculants, further amplify this effect by solubilizing nutrients like phosphorus and potassium, which are otherwise less mobile in the soil (Prasad et al., 2017). These processes create a more nutrient-rich rhizosphere, supporting robust plant growth and development. Additionally, the combination of (BOFs)with biochar can further boost CEC, as biochar’s porous structure provides additional sites for nutrient adsorption and exchange.

Organic manure and (BOFs)are vital tools for improving rhizosphere properties. Their slow nutrient release patterns, contributions to, and enhancement of (CEC)make them indispensable for sustainable soil management. By fostering nutrient retention and cycling, these fertilizers support healthier plants, more resilient soils, and reduced dependency on synthetic chemical inputs.

### 3.2 Rhizosphere properties

The application of organic amendments, such as organic manure and (BOFs), significantly influences rhizosphere properties in both the short and long term (Bai et al., 2023). These effects vary based on the composition of the amendment, its rate of application, and environmental conditions. Understanding the temporal dynamics of these amendments is essential for optimizing their role in sustainable agriculture. In the short term, organic amendments induce rapid changes in the physical and chemical properties of the rhizosphere. One immediate effect is the release of nutrients as the organic materials decompose. This process provides an initial supply of essential elements such as nitrogen, phosphorus, and potassium. For example, nitrogen is released in plant-available forms like ammonium and nitrate, supporting early-stage plant growth. Additionally, organic amendments stimulate microbial activity in the rhizosphere by supplying easily degradable organic compounds. This microbial stimulation enhances enzymatic processes that accelerate nutrient cycling (Shu et al., 2022). Short-term applications of organic amendments also improve soil structure and water retention in the rhizosphere. The addition of organic matter reduces soil compaction, increases porosity, and improves aeration, which collectively facilitate root penetration and nutrient uptake. However, the rapid decomposition of organic material can temporarily result in nutrient imbalances, such as nitrogen immobilization, as microbes compete with plants for available nitrogen during the decomposition process (Wortman et al., 2017).

In contrast, the long-term application of organic amendments provides cumulative benefits that enhance the resilience and fertility of the rhizosphere. Over time, organic amendments increase the (SOM) content, which contributes to improved soil structure, enhanced water-holding capacity, and better aggregation. This accumulation of SOM also fosters a more stable microbial community in the rhizosphere, promoting processes such as nutrient mineralization and pathogen suppression. Furthermore, repeated applications of organic amendments enhance the soil’s (CEC), improving its ability to retain and supply nutrients to plant roots (Singh et al., 2022). Another critical long-term benefit is the role of organic amendments in carbon sequestration. The addition of organic material increases the stable carbon fraction in soils, contributing to the mitigation of greenhouse gas emissions and improving the soil’s ability to withstand environmental stresses such as drought and erosion. This carbon sequestration not only benefits soil health but also aligns with global efforts to combat climate change (Cui et al., 2023).

Overall, while short-term effects of organic amendments focus on immediate improvements in nutrient availability and microbial activity, the long-term benefits result in sustained enhancements to soil fertility, structure, and resilience. These temporal dynamics highlight the importance of consistent and strategic application of organic amendments to achieve long-term sustainability in agricultural systems. While organic fertilizers play a key role in nutrient cycling and microbial activity, the integration of biochar presents an opportunity to further enhance these effects. The following section explores biochar’s unique properties and mechanisms of action as a soil amendment.

### 3.3 Synergistic effects of bio-organic fertilizers with soil microbes

Bio-organic fertilizers, a combination of organic materials and beneficial microbial inoculants, have a profound impact on soil health and plant productivity. Their efficacy lies in their ability to foster a synergistic relationship with soil microbes, enhancing nutrient cycling, organic matter decomposition, and overall rhizosphere health (Freitas et al., 2020). These interactions are critical for creating a dynamic soil ecosystem that supports sustainable agricultural practices. One of the key mechanisms underlying the synergy between (BOFs)and soil microbes is the provision of a readily available energy source. The organic matter in (BOFs)serves as a substrate for microbial communities, stimulating their growth and activity. This stimulation promotes the proliferation of beneficial microbes such as nitrogen-fixing bacteria (*Rhizobium* and *Azotobacter*), phosphate-solubilizing bacteria, and mycorrhizal fungi. These microbes play vital roles in nutrient transformation and uptake, thereby improving plant growth and soil fertility (Kour et al., 2020). Nitrogen-fixing bacteria, such as Rhizobium and Azotobacter, convert nitrogen gas (N₂) from the atmosphere into ammonia (NH₃), which plants can absorb and use for growth. This process naturally enriches soil nitrogen levels without the need for synthetic fertilizers. This process not only reduces the dependency on synthetic nitrogen fertilizers but also improves soil nitrogen levels over time. Similarly, phosphate-solubilizing bacteria release inorganic phosphorus bound to soil particles, making it accessible to plant roots. This is particularly valuable in phosphorus-deficient soils, where the microbial activity induced by (BOFs)can significantly enhance phosphorus availability in the rhizosphere (Kimble and Madigan, 1992).

Mycorrhizal fungi, another critical component of (BOFs), establish symbiotic relationships with plant roots. These fungi extend the effective root surface area through their hyphal networks, improving the uptake of water and nutrients, particularly phosphorus and micronutrients. In return, plants provide carbohydrates derived from photosynthesis to sustain fungal growth. This mutualistic interaction enhances nutrient acquisition and improves plant tolerance to environmental stresses, such as drought and salinity (Fall et al., 2022). The introduction of (BOFs)also supports microbial diversity in the soil. The inoculation of beneficial microbes can outcompete pathogenic microorganisms, reducing the incidence of soil-borne diseases. This competitive exclusion, combined with the enhanced microbial activity, creates a more resilient rhizosphere ecosystem. Additionally, the enzymes produced by these microbes accelerate the decomposition of organic matter, releasing nutrients into plant-available forms and improving soil structure and fertility (Olaniyan et al., 2022). Moreover, (BOFs)work synergistically with existing soil microbial communities by enhancing their functionality. The microbial inoculants introduced through these fertilizers interact with indigenous soil microbes, fostering a more active and diverse microbial network. This interaction amplifies key processes such as carbon and nitrogen cycling, leading to long-term improvements in soil health (Han et al., 2022).

The synergistic effects of (BOFs)and soil microbes not only enhance nutrient availability but also contribute to sustainable soil management. By leveraging the natural processes mediated by soil microbes, (BOFs)reduce the need for synthetic chemical inputs and mitigate the environmental impacts associated with conventional agriculture. This synergy underscores the importance of integrating (BOFs)into modern farming systems to promote both productivity and ecological sustainability.

## 4 Limitations of organic fertilizers and the need for improvement in nutrient availability

Organic fertilizers, including (BOFs)and organic manure, offer numerous environmental and agronomic benefits. However, they also face several limitations that hinder their widespread application and efficacy in meeting crop nutrient demands. Addressing these challenges is critical for optimizing the use of organic fertilizers in modern agricultural systems (Gelaye, 2023). One of the primary limitations of organic fertilizers is their slow nutrient release. Unlike synthetic fertilizers, which provide nutrients in immediately available forms, organic fertilizers rely on microbial decomposition to mineralize nutrients into plant-usable forms. This process can delay nutrient availability, particularly during critical growth stages when plants require high nutrient inputs. This delay in nutrient release can reduce crop yields, particularly in high-intensity farming systems (Shaji et al., 2021). Another challenge is the variable nutrient composition of organic fertilizers, which is influenced by the source material, processing methods, and storage conditions. For example, the nutrient content of manure differs significantly depending on the type of livestock, feed quality, and manure management practices. This variability complicates the precise calculation of nutrient contributions and hinders effective nutrient management planning (Wu et al., 2022).

Organic fertilizers also have a low nutrient density compared to synthetic fertilizers, requiring large application volumes to meet crop nutrient requirements. This bulkiness increases transportation and application costs, particularly for large-scale agricultural operations. Furthermore, repeated use of certain organic fertilizers, such as manure, can lead to nutrient imbalances in the soil. For instance, excessive phosphorus accumulation can reduce the availability of micronutrients like zinc and iron, potentially harming plant growth (Herrera et al., 2022). A common short-term issue with organic fertilizers is nitrogen immobilization, where soil microbes temporarily consume available nitrogen during the decomposition of high-carbon organic materials. This microbial activity reduces nitrogen availability to plants, potentially causing nutrient deficiencies during early crop development stages (Zhang et al., 2023).

Additionally, organic fertilizers derived from animal waste or municipal compost may contain contaminants, such as heavy metals, pathogens, or residual antibiotics. These contaminants pose risks to soil health, crop safety, and overall environmental quality, highlighting the need for stringent processing and quality control measures.

### 4.1 Improving nutrient availability of organic fertilizers

Efforts to enhance the nutrient availability and overall efficiency of organic fertilizers have led to several innovative approaches. One promising solution is the integration of biochar with organic fertilizers. Biochar’s porous structure improves nutrient retention, reduces leaching, and ensures a more consistent nutrient supply in the rhizosphere (Badu Brempong and Addo-Danso, 2022). The use of microbial inoculants in (BOFs) has also shown significant potential. These fertilizers combine organic materials with beneficial microbes, such as nitrogen-fixing bacteria, phosphate-solubilizing bacteria, and mycorrhizal fungi, to enhance nutrient mineralization and uptake. For example, nitrogen-fixing bacteria convert atmospheric nitrogen into plant-available forms, while phosphate-solubilizing bacteria release bound phosphorus in the soil (Rehan et al., 2024).

Advanced composting techniques are another strategy to improve organic fertilizer performance. Controlled composting processes can stabilize nutrient content, minimize variability, and reduce contaminants, ensuring a safer and more reliable product. Similarly, precision application methods, such as banding or foliar application of organic extracts, align nutrient availability with plant demand, improving nutrient use efficiency and reducing wastage (Liu et al., 2022). In some cases, blending organic fertilizers with synthetic fertilizers can combine the immediate nutrient availability of synthetic inputs with the long-term soil health benefits of organic matter. Such integrated nutrient management systems can enhance productivity while maintaining soil fertility over time (Asif et al., 2024). Despite their limitations, organic fertilizers remain a cornerstone of sustainable agriculture due to their environmental and soil health benefits. Addressing challenges such as slow nutrient release, variability in composition, and nutrient imbalances requires innovative approaches, including biochar integration, microbial fortification, and advanced composting techniques. By improving nutrient availability and efficiency, these advancements can enhance the role of organic fertilizers in modern agricultural systems, reducing dependency on synthetic inputs and promoting long-term sustainability.

## 5 Biochar as a soil amendment: properties and mechanisms of action

Biochar is a stable, carbon-rich material produced through a process called pyrolysis, where organic biomass is heated in a low-oxygen or oxygen-free environment. This process leads to the thermal decomposition of biomass, resulting in three primary products: biochar, bio-oil, and syngas. Biochar, the solid product, is valued for its ability to improve soil properties such as structure, fertility, and carbon sequestration. Pyrolysis typically occurs at temperatures ranging between 300°C and 700°C, though the specific temperature used can influence the biochar’s characteristics, such as its porosity, surface area, and carbon stability. Higher pyrolysis temperatures (>500°C) generally produce biochar with more stable carbon and greater surface area, which enhances its potential for carbon sequestration and soil aeration. Conversely, lower pyrolysis temperatures (<400°C) produce biochar with higher nutrient content, which can be beneficial for soil fertility, particularly in nutrient-poor soils (De Bhowmick et al., 2019).

The pyrolysis process is highly influenced by the selected feedstock, as different materials yield biochar with varying properties. Feedstocks for biochar production can come from a variety of organic sources, each contributing distinct qualities to the biochar produced. Agricultural residues such as crop straw, rice husks, and corn stalks are commonly used for biochar production, resulting in biochar with relatively high nutrient content, particularly in terms of phosphorus and potassium (Chachar et al., 2024). This makes agricultural residue-based biochar particularly useful for enhancing soil fertility. Forestry by-products, including sawdust, wood chips, and bark, typically produce biochar with a high carbon content and greater structural stability, which is beneficial for improving soil aeration and promoting long-term soil health. These types of biochar are less nutrient-rich, making them more suitable for applications focused on improving soil structure and carbon storage, rather than providing immediate nutrients for plant growth (Tripathi et al., 2016).

Livestock manure, including cow, poultry, and other animal waste, is another important feedstock used in biochar production. Manure-based biochar is rich in nitrogen, phosphorus, and potassium, making it an excellent amendment for soils that are deficient in these nutrients. However, this biochar type requires careful processing to mitigate potential contaminants, such as pathogens or heavy metals, which may be present in the manure. Municipal solid waste, including green waste and yard trimmings, can also serve as a feedstock for biochar production. Though this feedstock helps recycle organic waste, it may contain contaminants such as plastics or heavy metals, requiring additional treatment to ensure the safety of the final biochar product (Khoshnevisan et al., 2021).The properties of biochar are influenced by both the feedstock used and the conditions under which it is produced. For example, biochar produced from wood-based feedstocks tends to have a higher surface area and greater structural stability, making it ideal for improving long-term soil health and for carbon sequestration. On the other hand, biochar derived from manure or agricultural residues often contains more nutrients, which can directly enhance soil fertility and microbial activity. In essence, the pyrolysis temperature and feedstock type dictate the physical and chemical properties of biochar, determining whether it is best suited for improving soil structure, increasing nutrient availability, or contributing to long-term carbon storage (Li et al., 2022).

Biochar production through pyrolysis is a flexible and adaptable process that can be tailored to achieve specific agricultural or environmental goals. By carefully selecting feedstocks and adjusting pyrolysis conditions, biochar can be produced to enhance soil fertility, improve soil structure, and sequester carbon, making it a valuable tool for sustainable agricultural practices and environmental management.

### 5.1 Physical and chemical properties of biochar

Biochar is renowned for its unique physical and chemical properties, which make it a highly effective soil amendment. These properties, such as high surface area, porosity, water-holding capacity, alkaline nature, and nutrient retention capabilities, play crucial roles in enhancing soil structure, fertility, and microbial activity (Karthik et al., 2020). One of the most notable physical properties of biochar is its high surface area and porosity. The pyrolysis process creates a highly porous structure within biochar, with extensive internal surface areas. These pores are beneficial for improving soil aeration, root penetration, and water retention. The porous nature of biochar also provides ample surface area for the adsorption and retention of water, which improves soil moisture retention in both sandy and loamy soils. As a result, biochar helps mitigate water stress in plants by enhancing the soil’s ability to hold water, which is particularly useful in arid and semi-arid regions (Leng et al., 2021).

In addition to improving water retention, biochar’s alkaline nature is another significant characteristic that influences soil chemistry. Biochar typically has a high pH (often ranging from 7.5 to 10), which can help neutralize acidic soils. The alkalinity of biochar can significantly raise the pH of acidic soils, thereby improving nutrient availability and enhancing plant growth. In soils with low pH, biochar acts as a liming agent, reducing the harmful effects of soil acidity on plant roots and promoting a more favorable environment for beneficial soil microorganisms (Razzaghi et al., 2020). The nutrient adsorption and retention properties of biochar are also key to its role as a soil amendment. Biochar has a remarkable ability to adsorb cations (positively charged ions) and anions (negatively charged ions), which are crucial for plant nutrition. This property is particularly beneficial in soils prone to nutrient leaching, as biochar helps retain essential nutrients such as nitrogen, phosphorus, potassium, calcium, and magnesium. By adsorbing these nutrients, biochar prevents their loss through leaching and improves their availability to plants over time. Furthermore, biochar’s (CEC) enhances its ability to hold and release nutrients in a form that plants can readily absorb, making it an excellent addition to nutrient-deficient soils (Hossain et al., 2020).

The chemical properties of biochar, including its ability to adsorb both organic and inorganic molecules, are determined by the feedstock used and the pyrolysis conditions. Biochar produced from different feedstocks may exhibit variations in nutrient content, pH, and surface chemistry. For instance, biochar made from wood-based feedstocks tends to have a lower pH and greater stability, while biochar produced from manure or agricultural residues may have higher nutrient content, particularly nitrogen and phosphorus, making it more suitable for improving soil fertility in the short term (Yaashikaa et al., 2020).

The physical and chemical properties of biochar—including its high surface area, porosity, alkaline nature, and nutrient retention capacity—make it an effective soil amendment. By improving soil structure, enhancing nutrient availability, and mitigating water stress, biochar has the potential to significantly contribute to sustainable agricultural practices, especially in regions facing challenges such as nutrient-poor soils, water scarcity, or soil acidity.

### 5.2 Mechanisms of biochar’s action in soil

Biochar exerts a range of beneficial effects on soil health through various mechanisms, contributing to improved soil structure, enhanced water retention, reduced nutrient leaching, and more efficient nutrient cycling. Additionally, biochar positively impacts soil microbial communities and plant health, making it a valuable soil amendment in sustainable agricultural systems (Wang et al., 2024). One of the primary mechanisms by which biochar improves soil is through the enhancement of soil structure and water retention. Biochar’s high porosity and surface area create a more open and aerated soil structure, which facilitates root penetration and gas exchange. This improved structure also contributes to enhanced soil aggregation, reducing soil compaction and increasing soil permeability. The porous nature of biochar enables it to act as a sponge, increasing the soil’s water-holding capacity, which is particularly beneficial in regions with irregular rainfall or in drought-prone areas. By retaining moisture in the soil, biochar helps maintain plant growth during dry periods and reduces the frequency of irrigation needed, making it an essential tool for water conservation in agriculture (Joseph et al., 2021).

Another important mechanism of biochar’s action is its ability to reduce nutrient leaching and improve nutrient cycling. The porous structure of biochar provides numerous sites for the adsorption and retention of nutrients, including essential macronutrients (nitrogen, phosphorus, potassium) and micronutrients. In nutrient-poor soils or in regions where heavy rainfall causes nutrient leaching, biochar can effectively trap nutrients and reduce their loss, ensuring their availability to plants over an extended period. Moreover, biochar enhances the (CEC) of soils, which increases the soil’s ability to retain and release cations such as calcium, magnesium, and potassium. This improves nutrient cycling by reducing nutrient loss and facilitating more efficient nutrient uptake by plants. As a result, biochar helps maintain long-term soil fertility, particularly in soils prone to nutrient depletion (Gao and DeLuca, 2022).

Furthermore, biochar has a significant impact on soil microbial communities and plant health. The porous and stable structure of biochar creates an ideal habitat for beneficial microorganisms, including bacteria, fungi, and actinomycetes, which play a crucial role in nutrient cycling and soil health. By providing a habitat for these microorganisms, biochar enhances microbial diversity and activity, particularly in the rhizosphere (the soil zone surrounding plant roots). This, in turn, supports the breakdown of organic matter, the transformation of nutrients into plant-available forms, and the suppression of harmful soil-borne pathogens. Biochar also helps to improve soil pH, particularly in acidic soils, which further promotes the growth and activity of beneficial microbes. In addition, biochar can enhance plant health by improving soil nutrient availability, reducing water stress, and protecting plants from harmful pathogens, leading to increased crop yield and resilience (Wang C, et al., 2021). Biochar improves soil health and fertility through several key mechanisms: enhancing soil structure and water retention, reducing nutrient leaching and promoting efficient nutrient cycling, and supporting soil microbial communities and plant health. These mechanisms collectively make biochar a powerful tool for sustainable agriculture, especially in soils affected by nutrient depletion, water scarcity, or poor microbial activity.

**Figure 2:**
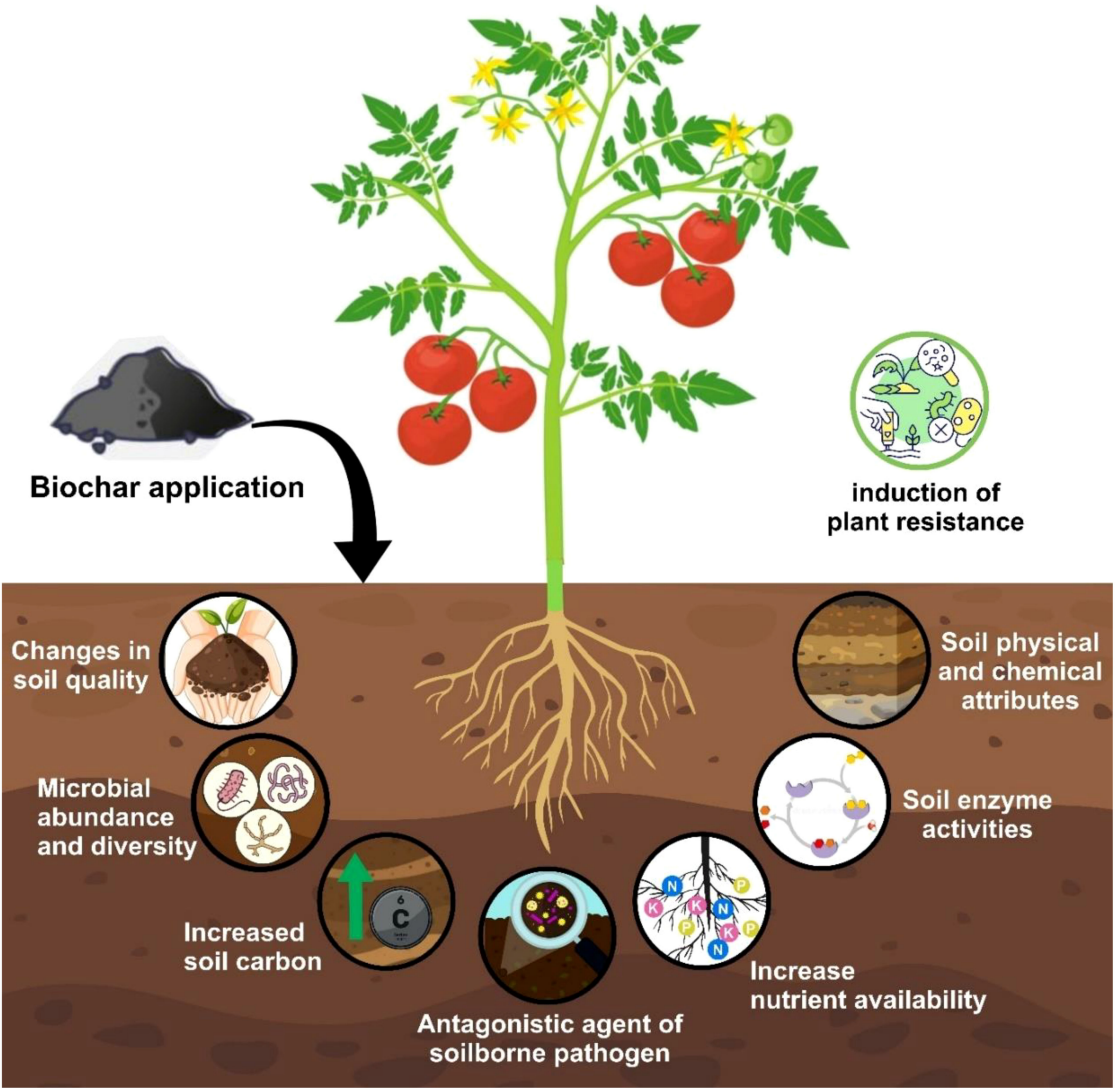
Effects of biochar application on soil properties and plant health. The schematic illustrates the benefits of biochar incorporation in the soil, including changes in soil quality, increased microbial abundance and diversity, and enhanced soil carbon content. Biochar also acts as an antagonistic agent against soilborne pathogens while improving soil physical and chemical properties, enzyme activities, and nutrient availability. Moreover, biochar contributes to the induction of plant resistance, ultimately supporting improved crop growth and productivity.

The application of biochar significantly influenced soil properties, microbial communities, and plant resistance mechanisms. Biochar amendments led to noticeable improvements in soil quality, including enhanced soil physical and chemical attributes and increased enzyme activity. The addition of biochar also contributed to greater microbial abundance and diversity, promoting beneficial interactions within the soil microbiome. A substantial increase in soil carbon content was observed, which is likely to improve soil fertility and structure. Furthermore, biochar exhibited antagonistic properties against soilborne pathogens, potentially reducing disease incidence. Nutrient availability was enhanced, facilitating improved plant growth and development. Additionally, biochar played a role in inducing plant resistance, suggesting its potential as a sustainable strategy to enhance crop resilience under various environmental conditions (**Figure 2**).

### 5.3 Biochar’s effect on enzyme activities in the rhizosphere

Soil enzymes play a critical role in nutrient cycling by facilitating the breakdown of organic matter and the transformation of nutrients into forms that plants can readily absorb. These enzymes, produced by soil microorganisms and plant roots, act as biological catalysts, accelerating chemical reactions in the rhizosphere. The activity of soil enzymes serves as an indicator of soil health and microbial activity, as they are essential for maintaining the balance of nutrients within the soil-plant system. Key enzymes involved in nutrient cycling include dehydrogenases, phosphatases, cellulases, proteases, and those related to the nitrogen cycle (Neemisha and Sharma, 2022). Dehydrogenases are central to the microbial oxidation of organic matter in the soil. These enzymes are involved in energy transfer within microbial cells and are indicative of overall microbial activity in the soil. Higher dehydrogenase activity reflects an active and healthy microbial community, which is critical for nutrient mineralization and (SOM)decomposition (Bogati and Walczak, 2022).

Phosphatases play a vital role in phosphorus cycling by hydrolyzing organic phosphorus compounds into inorganic phosphate, which is a form readily available to plants. Phosphorus is often a limiting nutrient in soils, and phosphatases help mobilize this nutrient, enhancing its availability in the rhizosphere. The activity of phosphatases is particularly important in soils amended with organic fertilizers or biochar, where phosphorus availability can be increased through microbial processes (Sekaran et al., 2020). Cellulases are responsible for breaking down cellulose, the most abundant organic polymer in plant residues. These enzymes decompose complex carbohydrates into simpler sugars, which can be further metabolized by soil microbes. The activity of cellulases contributes to the decomposition of organic matter, releasing nutrients such as carbon, nitrogen, and sulfur into the soil (Sharma, 2021).

Proteases are involved in the nitrogen cycle by breaking down proteins into smaller peptides and amino acids, which can then be further degraded into ammonium and nitrate by other microbial processes. Protease activity is essential for nitrogen mineralization, a process that ensures a steady supply of nitrogen to plants in soils amended with organic materials (Van Der Hoorn and Klemenčič, 2021). Enzymes related to the nitrogen cycle, such as nitrogenase, nitrate reductase, and urease, are integral to nitrogen transformations in the soil. Nitrogenase is responsible for biological nitrogen fixation, converting atmospheric nitrogen into ammonia, a form that plants can utilize. Nitrate reductase catalyzes the reduction of nitrate to nitrite, while urease hydrolyzes urea into ammonium. Together, these enzymes ensure the continuous cycling of nitrogen within the soil, supporting plant growth and development (Boon et al., 2020).

The activity of these enzymes is influenced by various soil properties, including pH, moisture content, organic matter availability, and microbial diversity. The addition of biochar to the soil can significantly enhance enzyme activities by improving these soil properties and providing a favorable habitat for microbial communities. For example, biochar’s ability to increase soil pH in acidic soils can enhance the activity of pH-sensitive enzymes such as phosphatases. Similarly, the high surface area and porosity of biochar create niches for microbial colonization, indirectly boosting enzyme production and activity (Fan et al., 2022).

Soil enzymes are indispensable for nutrient cycling in the rhizosphere, contributing to the decomposition of organic matter and the transformation of nutrients into plant-available forms. The interaction of biochar with these enzymatic processes has the potential to improve soil fertility and promote sustainable agricultural practices.

### 5.4 Short-term vs. long-term effects of biochar on enzyme activities

The application of biochar to soil influences enzyme activities in both the short term and the long term, enhancing nutrient cycling, organic matter decomposition, and overall soil health. These effects differ in magnitude and mechanisms, reflecting biochar’s interaction with (SOM), microbial communities, and environmental conditions (Liao et al., 2022).

In the short term, biochar stimulates enzyme production by providing a favorable environment for microbial activity, particularly when used in combination with organic manure amendments. The porous structure of biochar offers a habitat for microorganisms, allowing them to thrive and produce enzymes essential for nutrient cycling. For example, when organic manure is applied alongside biochar, the organic compounds in the manure serve as substrates for microbial activity, leading to increased production of enzymes such as dehydrogenases, phosphatases, and cellulases. Dehydrogenase activity, which is closely linked to microbial respiration and organic matter decomposition, typically shows a rapid increase after biochar application, reflecting enhanced microbial metabolic processes. Similarly, phosphatases involved in phosphorus cycling exhibit higher activity due to biochar’s capacity to buffer soil pH, particularly in acidic soils, making phosphorus more accessible for enzymatic transformation (Lopes et al., 2021). Another short-term effect of biochar is its ability to mitigate nutrient losses by adsorbing and retaining nutrients released from organic amendments. This nutrient retention prevents leaching and provides a steady supply of substrates for enzyme-mediated reactions, further stimulating enzymatic activity. These immediate benefits contribute to enhanced nutrient availability and plant growth during the early stages of biochar application (Das et al., 2021).

In the long term, biochar’s effects on enzyme activities become more stable and sustained as it integrates into the soil matrix and interacts with the existing (SOM)and microbial community. Over time, biochar contributes to the accumulation of stable organic matter in the soil, creating a consistent supply of substrates for enzymatic reactions. This stability promotes a resilient soil ecosystem where enzyme activities are maintained at elevated levels, supporting continuous nutrient cycling. For example, cellulase activity remains enhanced due to the long-term decomposition of plant residues and the stabilization of organic matter facilitated by biochar. Protease activity, which is critical for nitrogen mineralization, also benefits from the gradual release of nitrogen compounds adsorbed onto biochar surfaces (Enaime and Lübken, 2021).

Biochar’s influence on enzyme activities in the long term is also mediated by its impact on soil physical and chemical properties. By improving soil structure, increasing water retention, and enhancing cation exchange capacity, biochar creates an optimal environment for enzyme function. Additionally, the long-lasting effect of biochar on soil pH stabilization supports the activity of pH-sensitive enzymes, such as phosphatases, ensuring efficient phosphorus cycling over time (Gunarathnea et al., 2022). When biochar is applied alongside organic manure, the long-term synergistic effects are particularly pronounced. Organic manure provides a continuous supply of degradable organic matter, while biochar stabilizes the nutrient pool and supports microbial populations responsible for enzyme production. This combination leads to sustained enzyme activities that enhance soil fertility and resilience (Kul et al., 2022).

The short-term effects of biochar on enzyme activities are driven by its immediate interactions with soil microbes and organic amendments, resulting in a rapid increase in enzymatic processes. In the long term, biochar contributes to a stable and enriched soil environment that supports continuous enzyme activity and nutrient cycling. These dual benefits underscore the importance of biochar as a sustainable amendment for improving soil health and promoting long-term agricultural productivity.

### 5.5 Mechanisms of biochar in enhancing enzyme activities

Biochar enhances enzyme activities in soil through multiple mechanisms that involve its influence on microbial communities, soil properties, and enzyme stability. These mechanisms contribute to improved nutrient cycling, organic matter decomposition, and overall soil health, making biochar an effective amendment for promoting sustainable agriculture (Pokharel et al., 2020). One of the primary mechanisms by which biochar boosts soil enzyme activity primarily by improving conditions for beneficial microbes. Its porous structure provides a habitat for microbes to colonize, while also storing water and nutrients they need to thrive. As microbial populations increase, so does their production of enzymes that support nutrient cycling. The porous structure of biochar provides an ideal habitat for soil microorganisms, offering protection and microenvironments that support their growth and survival. These pores create physical spaces where microbes can colonize, particularly in soils that lack sufficient aggregation or organic matter. By improving microbial habitat, biochar indirectly promotes the production of soil enzymes, which are largely secreted by microbial communities. Furthermore, biochar acts as a source of carbon for some microbial species, stimulating their metabolic processes and increasing their enzymatic output. For example, dehydrogenase activity, a key indicator of microbial respiration, often shows significant improvement following biochar application due to the increased microbial biomass supported by biochar (Deshoux et al., 2023).

Biochar also enhances enzyme activity by stabilizing enzymes in the soil, thereby extending their functional lifespan. Many soil enzymes are adsorbed onto the surfaces of soil particles or organic matter, and biochar provides an additional surface for this adsorption. The high surface area and porous nature of biochar allow it to act as a reservoir for enzymes, protecting them from degradation by environmental factors such as extreme pH, temperature fluctuations, or microbial consumption. By binding enzymes to its surface, biochar prevents their denaturation and maintains their catalytic function for extended periods. This stabilization effect is particularly beneficial for enzymes involved in nutrient cycling, such as phosphatases and cellulases, which play crucial roles in making phosphorus and carbon available for plant uptake (Peiris et al., 2018). Another way biochar stabilizes enzymes is through its impact on soil chemical properties. By buffering soil pH, biochar creates a more favorable environment for pH-sensitive enzymes, ensuring their optimal activity. For instance, in acidic soils, biochar’s alkalinity neutralizes excess acidity, allowing enzymes like phosphatases to function more efficiently. Similarly, biochar’s ability to improve soil moisture retention creates a more consistent hydration environment, protecting enzymes from desiccation and maintaining their activity over time (Khan et al., 2024).

In addition to stabilizing existing enzymes, biochar indirectly enhances enzymatic processes by reducing nutrient losses in the soil. By adsorbing and retaining nutrients such as nitrogen and phosphorus, biochar ensures a steady supply of substrates for enzymatic reactions. This interaction amplifies nutrient cycling processes, as enzymes have access to more consistent concentrations of reactants, leading to increased overall soil fertility (Alam et al., 2020). Biochar enhances enzyme activities in soil through its dual role in increasing microbial biomass and activity and stabilizing enzymes, ensuring their prolonged functionality. These mechanisms not only improve nutrient cycling but also contribute to the resilience and sustainability of soil ecosystems, underscoring the importance of biochar as a soil amendment in modern agricultural practices.

### 5.6 The effect of biochar on enzymatic efficiency and nutrient mineralization in soils amended with organic fertilizers

The application of biochar, particularly in combination with organic fertilizers, significantly enhances enzymatic efficiency and nutrient mineralization in soils. This synergistic effect stems from biochar’s ability to improve soil physical and chemical properties, provide a favorable environment for microbial communities, and optimize the availability of substrates for enzymatic reactions. These processes collectively contribute to improved nutrient cycling and soil fertility (Javeed et al., 2023). Biochar enhances enzymatic efficiency by creating conditions that promote optimal enzyme activity. Its porous structure and large surface area provide microhabitats for soil microorganisms, which are the primary producers of soil enzymes. When organic fertilizers are added alongside biochar, the decomposable organic matter serves as a substrate for microbial metabolism, stimulating the production of key enzymes involved in nutrient cycling, such as phosphatases, cellulases, and proteases. The close interaction between biochar, organic matter, and microbes leads to higher enzymatic efficiency by ensuring that enzymes are produced in proximity to their substrates, thereby reducing energy losses and increasing reaction rates (Bolan S, et al., 2023).

The buffering capacity of biochar also contributes to enzymatic efficiency by stabilizing soil pH. Many soil enzymes, including phosphatases and ureases, are highly sensitive to pH fluctuations. Biochar’s alkaline nature neutralizes acidic soils, creating a more conducive environment for enzymatic activity. This effect is particularly pronounced in soils amended with organic fertilizers, which may temporarily acidify the rhizosphere during decomposition. By maintaining a stable pH, biochar ensures that enzymes can function at their optimal activity levels, enhancing the overall efficiency of nutrient mineralization processes (Arwenyo et al., 2023). Nutrient mineralization is a critical process in which organic nutrients are converted into inorganic forms that plants can readily absorb. Biochar accelerates this process by interacting with both organic fertilizers and soil microorganisms. For example, organic fertilizers provide organic nitrogen in the form of proteins and amino acids, which are broken down by proteases into ammonium and nitrate. Biochar enhances this process by retaining the mineralized nitrogen within its porous structure, preventing nutrient losses through leaching or volatilization. This retention not only ensures a steady supply of nitrogen to plants but also supports the activity of nitrogen-cycle enzymes, such as ureases and nitrases, which further facilitate nitrogen transformations (Li et al., 2020).

Similarly, biochar enhances phosphorus mineralization by providing adsorption sites that retain phosphate ions, reducing their immobilization in the soil. This is particularly beneficial in phosphorus-deficient or calcareous soils, where biochar prevents the precipitation of phosphorus with calcium or aluminum ions, making it more available for enzymatic activity. Phosphatases, which hydrolyze organic phosphorus compounds into plant-available phosphate, show increased activity in soils amended with biochar and organic fertilizers due to the improved availability of organic and inorganic phosphorus substrates (Ghodszad et al., 2021). The combination of biochar and organic fertilizers also promotes the mineralization of carbon and other macronutrients. Cellulases, which break down cellulose into simple sugars, exhibit enhanced activity in biochar-amended soils, as biochar provides a stable environment for microbial communities and retains decomposed organic matter. This results in a more efficient release of carbon and energy for microbial growth, further driving nutrient cycling in the rhizosphere (Lenka et al., 2022).

The addition of biochar to soils amended with organic fertilizers enhances enzymatic efficiency and nutrient mineralization by improving microbial activity, stabilizing soil conditions, and optimizing substrate availability. These interactions contribute to improved soil fertility and nutrient use efficiency, highlighting the value of integrating biochar with organic fertilizers in sustainable agricultural practices.

## 6 Short- and long-term effects of biochar and organic fertilizer application

The substitution of chemical fertilizers with biochar and organic fertilizers, such as organic manure, induces significant short-term changes in rhizosphere properties and enzymatic activities. These immediate effects contribute to improved nutrient availability, soil health, and microbial activity, providing a strong foundation for sustainable agricultural practices (Ren et al., 2021). One of the most notable short-term impacts is the immediate improvement in rhizosphere properties and enzyme activity following the application of biochar and organic manure. Biochar’s porous structure enhances soil aeration, water retention, and aggregation, while organic manure provides readily decomposable organic matter that stimulates microbial activity. Together, these amendments create a favorable environment for enzymatic processes critical to nutrient cycling. For instance, dehydrogenase activity, an indicator of microbial respiration, increases rapidly due to the availability of fresh organic substrates. Similarly, phosphatases and proteases exhibit enhanced activity as microbes decompose organic matter, releasing phosphorus and nitrogen in plant-available forms (Sandhu et al., 2019).

Another important short-term effect is enhanced nutrient retention and reduced leaching. Biochar acts as a reservoir for essential nutrients by adsorbing and retaining cations and anions such as ammonium, nitrate, and phosphate. This nutrient retention minimizes leaching losses, particularly in sandy or highly permeable soils. When combined with organic manure, biochar prevents the rapid loss of nutrients released during manure decomposition, ensuring that they remain available in the rhizosphere for plant uptake. This interaction not only improves nutrient use efficiency but also reduces the environmental risks associated with nutrient runoff, such as water eutrophication (Limwikran et al., 2019). Increased enzyme activities in the rhizosphere are another immediate benefit of biochar and organic manure application. The organic matter in manure serves as a substrate for microbial metabolism, stimulating the production of enzymes involved in organic matter decomposition and nutrient cycling. Biochar enhances this process by providing a stable habitat for microbes and protecting enzymes from denaturation. For example, cellulase activity increases as microbes break down plant residues into simpler carbohydrates, while urease activity rises due to the hydrolysis of urea into ammonium. The buffering effect of biochar on soil pH further supports the activity of pH-sensitive enzymes such as phosphatases, which are essential for phosphorus availability in acidic soils (Wang Q, et al., 2021).

The short-term effects of biochar and organic fertilizer substitution include immediate improvements in rhizosphere properties, enhanced nutrient retention, and increased enzyme activities. These changes provide a robust starting point for sustainable nutrient management, contributing to both plant productivity and environmental protection (Nguyen et al., 2018). One of the most significant long-term benefits of biochar is its role as a carbon sequestration agent. Biochar is composed of stable carbon fractions that resist decomposition, allowing it to remain in the soil for decades or even centuries. This persistence makes biochar a powerful tool for mitigating climate change by reducing atmospheric carbon dioxide levels. As a carbon sink, biochar not only offsets greenhouse gas emissions but also enhances (SOM)content, which improves soil structure, water retention, and nutrient-holding capacity. Over time, the integration of biochar into the soil matrix fosters the development of more stable soil aggregates, reducing erosion and enhancing the physical integrity of the soil (Murtaza et al., 2021).

The sustainability of biochar-amended organic fertilizers lies in their ability to maintain soil fertility and ecosystem services over the long term. Biochar improves the efficiency and longevity of organic fertilizers by reducing nutrient losses through leaching and volatilization. Its porous structure and high (CEC) retain nutrients such as nitrogen, phosphorus, and potassium, ensuring their gradual release and availability to plants. This sustained nutrient supply reduces the need for repeated fertilizer applications, lowering input costs and minimizing environmental pollution (Ayaz et al., 2021). Additionally, the long-term application of biochar-amended organic fertilizers supports the development of diverse and resilient microbial communities in the rhizosphere. These microbes play critical roles in nutrient cycling, organic matter decomposition, and disease suppression. By providing a stable habitat and enhancing nutrient availability, biochar fosters microbial activity that sustains soil fertility over time. This microbial-driven nutrient cycling enhances the provision of ecosystem services such as carbon storage, water filtration, and biodiversity conservation (Yuan et al., 2023).

Biochar also contributes to soil pH stabilization, particularly in acidic soils, where it neutralizes acidity and improves the availability of essential nutrients like phosphorus and magnesium. This effect persists over the long term, reducing the need for external pH amendments and creating a more balanced soil environment that supports plant growth and soil health (Bolan N, et al., 2023). The long-term application of biochar and organic fertilizers has profound and sustained impacts on soil health, carbon sequestration, and ecosystem services. Over time, these amendments contribute to the resilience and fertility of agricultural soils, supporting sustainable crop production and environmental sustainability. The long-term effects of biochar and organic fertilizers are transformative for soil health and agricultural sustainability. By acting as a carbon sequestration agent, biochar contributes to climate change mitigation, while its synergy with organic fertilizers enhances nutrient retention, microbial activity, and soil resilience. These enduring benefits underscore the value of biochar-amended organic fertilizers in maintaining soil fertility and ecosystem services, providing a sustainable foundation for future agricultural practices.

Beyond soil fertility and microbial activity, the integration of biochar and organic fertilizers also holds significant promise for environmental protection. In the next section, we examine the broader implications of this practice, including its potential to mitigate greenhouse gas emissions and reduce nutrient runoff.

### 6.1 Synergistic effects of biochar and organic fertilizers for sustainable nutrient cycling and soil health

The combination of biochar and organic fertilizers offers a synergistic approach to enhancing nutrient cycling and improving soil health, making it an integral part of sustainable agricultural practices. This synergy arises from the complementary properties of biochar and organic fertilizers, which together address nutrient availability, soil structure, and microbial activity in a holistic manner (Kocsis et al., 2022). Biochar’s porous structure and high (CEC) make it an excellent medium for retaining nutrients released from organic fertilizers. Organic fertilizers, such as manure or compost, provide a rich source of nutrients and organic matter, but they are prone to losses through leaching and volatilization. When applied in conjunction with biochar, these losses are significantly reduced, as biochar adsorbs and retains essential nutrients like nitrogen, phosphorus, and potassium. This retention ensures a gradual release of nutrients, aligning with plant demand and improving nutrient use efficiency. The result is a more sustainable nutrient cycling process, reducing the frequency of fertilizer applications and minimizing environmental pollution (Munera-Echeverri et al., 2018).

The combination of biochar and organic fertilizers also improves soil structure and water retention, both of which are critical for nutrient cycling and plant health. Biochar enhances soil porosity, aeration, and aggregation, while organic fertilizers contribute organic matter that binds soil particles together. Together, they create a more stable and permeable soil environment that facilitates root penetration, microbial activity, and nutrient transport. The improved water-holding capacity of biochar-amended soils further supports the decomposition of organic matter and the enzymatic processes necessary for nutrient mineralization (Akhtyamova et al., 2019). A key aspect of the synergy between biochar and organic fertilizers is their impact on microbial activity and diversity. Organic fertilizers provide a source of carbon and nutrients that fuel microbial metabolism, while biochar offers a habitat that protects microbes from environmental stressors. This combination fosters a thriving microbial community that drives nutrient cycling processes, such as nitrogen fixation, phosphorus solubilization, and organic matter decomposition. Enhanced microbial activity in the rhizosphere leads to higher enzymatic efficiency, supporting the transformation of organic nutrients into plant-available forms (Yan et al., 2023). Furthermore, the buffering capacity of biochar enhances the effectiveness of organic fertilizers by stabilizing soil pH, particularly in acidic soils. Many nutrients, including phosphorus and magnesium, become more available at neutral pH levels. By neutralizing soil acidity, biochar creates an environment that maximizes the nutrient contributions of organic fertilizers and supports microbial processes critical to nutrient cycling (Soil PH and PH Buffering, 2020).

The long-term application of biochar and organic fertilizers contributes to soil carbon sequestration and the buildup of stable organic matter, ensuring the sustained provision of ecosystem services. This combination not only improves soil fertility but also enhances soil resilience to stresses such as drought, salinity, and erosion. The integration of biochar and organic fertilizers into agricultural systems represents a sustainable strategy for maintaining soil health, improving crop productivity, and reducing environmental impacts (Saidia, 2023). The synergistic effects of biochar and organic fertilizers optimize nutrient cycling, enhance microbial activity, and improve soil structure and fertility. This powerful combination supports sustainable agriculture by reducing nutrient losses, minimizing the reliance on synthetic inputs, and promoting the long-term health and productivity of soils **Table 1**.

**Table 1:** Effects of biochar on soil properties.

Soil Property	Biochar Effect	Supporting Detail
Soil Structure	Improves aggregation, reduces compaction	Enhances porosity and water infiltration
Water Retention	Increases moisture-holding capacity	Especially beneficial in sandy or arid soils
pH Regulation	Raises pH in acidic soils	Acts as a liming agent
Cation Exchange Capacity (CEC)	Enhances nutrient retention and availability	Improves efficiency of nitrogen, potassium, and calcium use
Microbial Habitat	Provides physical space for microbial colonization	Boosts microbial biomass and enzyme activity
Soil Organic Matter (SOM)	Promotes long-term stabilization of organic carbon	Supports carbon sequestration

## 7 Environmental implications of using biochar with organic fertilizers

Biochar has emerged as a critical tool for addressing climate change due to its remarkable ability to sequester carbon in soils over long periods. Produced through the pyrolysis of organic biomass in a low-oxygen environment, biochar stabilizes carbon that would otherwise be released into the atmosphere as carbon dioxide (CO₂) during natural decomposition or combustion. When applied to soils, biochar contributes to long-term carbon storage, reducing greenhouse gas emissions and mitigating the impacts of climate change (Kurniawan et al., 2023). The carbon in biochar is highly resistant to microbial degradation due to its aromatic structure, which results from the high-temperature pyrolysis process. This structural stability allows biochar to persist in soils for centuries or even millennia, effectively locking away carbon and acting as a carbon sink. Unlike organic fertilizers, which release carbon relatively quickly as organic matter decomposes, biochar retains its carbon content, making it an effective amendment for reducing the atmospheric CO₂ concentration (Fluturim and Luqman, 2023).

When used in conjunction with organic fertilizers, biochar enhances carbon sequestration by improving (SOM)stability. Organic fertilizers provide a source of labile carbon that fuels microbial activity and nutrient cycling, while biochar stabilizes the more recalcitrant fractions of organic matter. This interaction reduces the rate of carbon mineralization, ensuring that a larger proportion of organic carbon remains in the soil as humus or biochar-stabilized compounds. This synergy not only improves soil fertility but also contributes to long-term carbon storage, creating a dual benefit for agriculture and climate mitigation (Ndoung et al., 2021). Additionally, biochar indirectly reduces greenhouse gas emissions by improving soil properties and nutrient use efficiency. Its porous structure and high reduce nitrogen losses through volatilization and leaching, mitigating the release of nitrous oxide (N₂O), a potent greenhouse gas. Furthermore, biochar’s ability to stabilize soil pH and improve microbial activity supports the reduction of methane (CH₄) emissions from waterlogged or anaerobic soils. These combined effects make biochar a multifaceted solution for lowering the carbon footprint of agricultural practices (Fang et al., 2024).

The scalability of biochar production adds to its potential as a climate mitigation strategy. Biochar can be produced from a wide range of biomass feedstocks, including agricultural residues, forestry by-products, and municipal organic waste, offering an opportunity to repurpose waste materials into a valuable soil amendment. By integrating biochar into agricultural systems alongside organic fertilizers, carbon sequestration efforts can be aligned with improvements in soil health and crop productivity, creating a sustainable cycle of carbon management (Bibi and Rahman, 2023). The carbon sequestration potential of biochar lies in its ability to stabilize organic carbon in soils over long time scales, significantly mitigating climate change. When paired with organic fertilizers, biochar further enhances soil carbon storage and reduces greenhouse gas emissions, making it a vital component of sustainable agricultural and environmental management strategies.

**Figure 3:**
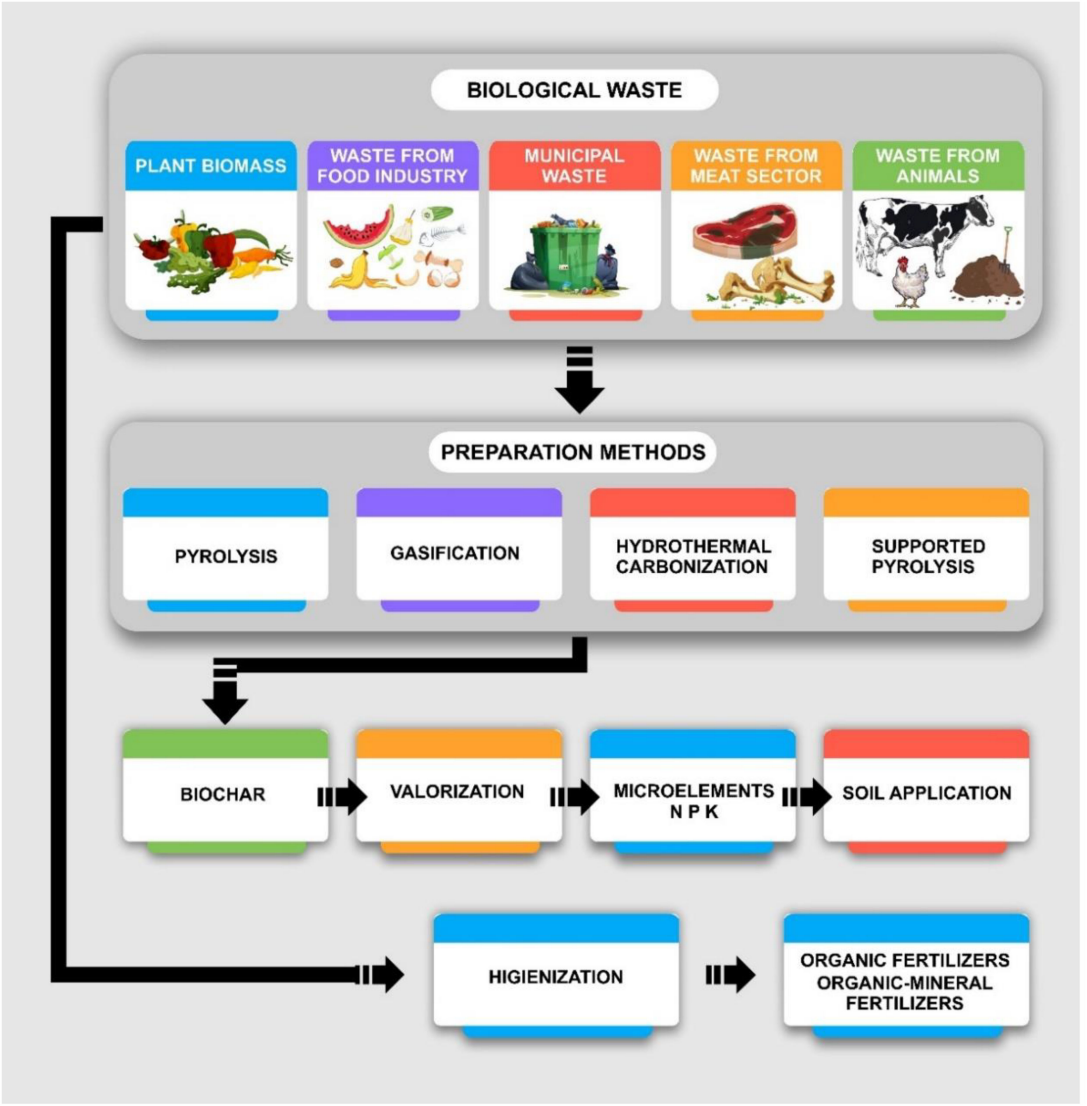
Schematic representation of biological waste transformation into biochar and organic fertilizers. Various sources of biological waste, including plant biomass, food industry waste, municipal waste, meat sector waste, and animal waste, undergo preparation through pyrolysis, gasification, hydrothermal carbonization, or supported pyrolysis. The resulting biochar is processed through valorization, supplemented with microelements (NPK), and applied to soil. Hygienization ensures the safe production of organic and organic-mineral fertilizers, contributing to sustainable waste management and soil improvement.

The transformation of biological waste into valuable agricultural products involves multiple stages, starting with the collection of diverse organic waste sources, including plant biomass, food industry waste, municipal waste, meat sector waste, and animal waste. These biological wastes undergo different preparation methods such as pyrolysis, gasification, hydrothermal carbonization, and supported pyrolysis to produce biochar. The resulting biochar undergoes further processes including valorization and enrichment with essential microelements like nitrogen (N), phosphorus (P), and potassium (K), making it suitable for soil application. Additionally, a hygienization step ensures the safe and effective utilization of biochar-derived materials. This process ultimately contributes to the production of organic and organic-mineral fertilizers, enhancing soil fertility and promoting sustainable agricultural practices (**Figure 3**).

### 7.1 Biochar and greenhouse gas reduction

Biochar plays a significant role in mitigating greenhouse gas (GHG) emissions from agricultural soils, particularly through the reduction of nitrous oxide (N₂O) and methane (CH₄) emissions. These gases are major contributors to global warming, with nitrous oxide having a global warming potential nearly 300 times that of carbon dioxide (CO₂), and methane being approximately 25 times more potent. The integration of biochar into soils, especially in combination with organic fertilizers, offers a promising strategy for reducing the emissions of these harmful gases (Shoudho et al., 2024). One of the primary ways biochar reduces nitrous oxide emissions is by improving soil nitrogen dynamics. Nitrous oxide is predominantly produced in soils through microbial processes such as nitrification and denitrification, which are influenced by soil aeration, moisture, and pH. Biochar’s porous structure enhances soil aeration by increasing pore space and reducing waterlogging, thereby limiting the anaerobic conditions that favor denitrification and subsequent N₂O production. Additionally, biochar’s high (CEC) improves nitrogen retention in the soil by adsorbing ammonium (NH₄^+^) and nitrate (NO₃⁻) ions, reducing the availability of nitrogen for microbial processes that generate nitrous oxide. This retention also minimizes nitrogen leaching, further reducing the risk of nitrous oxide emissions (Edwards et al., 2018).

Biochar’s pH buffering capacity is another critical factor in reducing nitrous oxide emissions. Many soils, particularly those amended with organic fertilizers, tend to become acidic over time due to the release of organic acids during decomposition. Biochar’s alkaline nature neutralizes soil acidity, creating a more favorable environment for microbial activity that promotes complete denitrification, which converts nitrate into harmless nitrogen gas (N₂) rather than nitrous oxide. This buffering effect ensures that nitrogen cycling processes are more efficient, reducing the accumulation of intermediates such as nitrous oxide (Rees et al., 2020). In the case of methane emissions, biochar is particularly effective in reducing CH₄ production in waterlogged or anaerobic soils, such as paddy fields or poorly drained agricultural lands. Methane is generated by methanogenic archaea during the decomposition of organic matter under anaerobic conditions. By improving soil aeration, biochar reduces the prevalence of anaerobic microenvironments, thereby inhibiting the activity of methanogens. Furthermore, biochar’s ability to adsorb labile organic carbon the primary substrate for methanogenesis—limits the availability of carbon for methane production, effectively reducing CH₄ emissions at their source (Kaur et al., 2023).

The interaction between biochar and soil microbial communities also contributes to lower methane emissions. Biochar supports the growth of methanotrophic bacteria, which consume methane and oxidize it into less harmful compounds. This microbial synergy further mitigates methane emissions, particularly in soils where biochar enhances microbial diversity and activity (Li et al., 2020). When combined with organic fertilizers, biochar amplifies its ability to reduce GHG emissions. Organic fertilizers, while beneficial for nutrient cycling and soil fertility, often contribute to increased emissions of nitrous oxide and methane due to their high nitrogen and carbon content. Biochar mitigates these emissions by stabilizing nitrogen and carbon in the soil, preventing their rapid release into the atmosphere. This synergistic effect makes the use of biochar-amended organic fertilizers an effective strategy for reducing the environmental footprint of agricultural practices (Xiong et al., 2022).

In conclusion, biochar significantly reduces soil emissions of nitrous oxide and methane by improving soil aeration, buffering pH, stabilizing nutrients, and supporting beneficial microbial communities. These mechanisms, combined with biochar’s interaction with organic fertilizers, highlight its potential as a sustainable solution for mitigating greenhouse gas emissions in agricultural systems.

### 7.2 Biochar’s role in reducing nutrient runoff

Nutrient runoff from agricultural fields is a major contributor to water pollution, leading to issues such as eutrophication, algal blooms, and the degradation of aquatic ecosystems. Biochar, when used as a soil amendment, plays a critical role in mitigating these impacts by reducing nutrient leaching and improving nutrient retention in soils. Its unique physical and chemical properties make it an effective tool for enhancing water quality while promoting sustainable nutrient management in agriculture (Quill et al., 2024). One of the primary mechanisms through which biochar reduces nutrient leaching is its high (CEC). Biochar’s porous structure and negatively charged surfaces adsorb positively charged ions such as ammonium (NH₄^+^), potassium (K^+^), and calcium (Ca²^+^), preventing them from being washed away during heavy rainfall or irrigation. This nutrient retention ensures that these essential nutrients remain in the soil, available for plant uptake, rather than contaminating nearby water bodies. Similarly, biochar’s ability to adsorb anions such as nitrate (NO₃⁻) and phosphate (PO₄³⁻) further reduces the loss of nutrients that are highly mobile in soil solutions (Lu et al., 2024).

In addition to nutrient adsorption, biochar enhances soil structure and water-holding capacity, which indirectly contributes to reducing nutrient runoff. By improving soil aggregation and porosity, biochar increases the soil’s ability to retain water, slowing down the movement of water through the soil profile. This decreased water flow reduces the likelihood of nutrient transport into surface and groundwater systems. As a result, biochar-amended soils exhibit lower rates of nutrient leaching, even under conditions of high precipitation or excessive irrigation (Lebrun et al., 2024). Biochar also interacts synergistically with organic fertilizers to prevent water pollution. Organic fertilizers, while beneficial for soil fertility, often release nutrients rapidly upon decomposition, increasing the risk of leaching and runoff. Biochar stabilizes these nutrients by adsorbing and slowly releasing them, aligning nutrient availability with plant demand. This slow-release mechanism not only improves nutrient use efficiency but also reduces the risk of nutrient losses into adjacent waterways (Lebrun et al., 2024).

The environmental benefits of biochar in reducing nutrient runoff extend to its role in preventing eutrophication. Excess nutrients, particularly nitrogen and phosphorus, are the primary drivers of eutrophication in aquatic ecosystems. When these nutrients leach into water bodies, they stimulate the overgrowth of algae, depleting oxygen levels and harming aquatic life. By retaining these nutrients in the soil, biochar minimizes their entry into rivers, lakes, and coastal waters, protecting aquatic ecosystems and maintaining water quality (Lebrun et al., 2024). Biochar’s effectiveness in mitigating nutrient runoff has been demonstrated across various soil types and agricultural systems. In sandy soils, which are particularly prone to nutrient leaching due to their low retention capacity, biochar significantly enhances nutrient adsorption and reduces losses. Similarly, in regions with high rainfall, biochar helps stabilize nutrients and prevents them from being carried away by surface runoff (Alessandrino, 2024).

Biochar plays a vital role in reducing nutrient leaching and preventing water pollution by improving nutrient retention, stabilizing organic fertilizers, and enhancing soil water-holding capacity. Its ability to mitigate nutrient runoff not only protects water quality but also promotes more efficient nutrient management, contributing to the sustainability of agricultural practices and the preservation of aquatic ecosystems.

### 7.3 Sustainable agriculture: biochar as part of integrated soil fertility management strategies for sustainable crop production

Biochar has emerged as a cornerstone in Integrated Soil Fertility Management (ISFM) strategies, offering a sustainable approach to enhancing crop production while maintaining soil health. ISFM combines organic amendments, mineral fertilizers, and improved soil management practices to optimize nutrient availability and use efficiency. Biochar’s unique properties make it a valuable component of ISFM, addressing key challenges in modern agriculture, such as soil degradation, nutrient losses, and climate change (Rahmiaty, 2021). One of biochar’s primary contributions to ISFM is its ability to improve nutrient retention and cycling. Biochar enhances the (CEC) of soils, enabling the retention of essential nutrients such as nitrogen, phosphorus, and potassium. This property reduces nutrient leaching and aligns nutrient availability with crop demand, minimizing fertilizer losses and improving nutrient use efficiency. When integrated with organic fertilizers and mineral inputs, biochar acts as a stabilizing agent, ensuring that nutrients are released gradually into the soil, thereby supporting sustained crop growth and reducing the need for frequent fertilization (Qian et al., 2015).

Biochar also improves soil structure and water-holding capacity, which are critical for sustainable crop production. Its porous structure enhances soil aeration and root penetration, while its ability to retain water helps mitigate the effects of drought and irregular rainfall. By improving soil physical properties, biochar creates a more resilient agricultural system capable of maintaining productivity under varying environmental conditions (Yadav and Singh, 2023). These benefits are particularly significant in regions with degraded soil or erratic climates, where biochar helps restore soil fertility and supports stable crop yields. As part of ISFM, biochar contributes to (SOM)accumulation, enhancing soil fertility over the long term. The application of biochar alongside organic fertilizers, such as compost or manure, fosters the buildup of stable organic carbon fractions in the soil. This not only improves soil nutrient cycling but also enhances soil resilience to erosion, salinity, and compaction. The integration of biochar with crop residues and cover cropping further amplifies its benefits, creating a synergistic effect that sustains soil health and productivity (Jia et al., 2024).

Biochar’s role in mitigating greenhouse gas emissions adds another dimension to its value in ISFM strategies. By stabilizing nitrogen in the soil and reducing nitrous oxide emissions, biochar lowers the environmental impact of agricultural practices. Its carbon sequestration potential also contributes to climate change mitigation, aligning sustainable crop production with global environmental goals (He et al., 2024). The economic feasibility of biochar within ISFM strategies is enhanced by its versatility and adaptability to local resources. Biochar can be produced from a wide range of feedstocks, including agricultural residues and forestry by-products, making it accessible to smallholder farmers and large-scale agricultural operations alike. Its long-lasting effects on soil health and nutrient availability reduce the dependency on synthetic fertilizers, lowering input costs and improving the profitability of farming systems over time (Afshar and Mofatteh, 2024).

In conclusion, biochar is a transformative component of Integrated Soil Fertility Management strategies, contributing to sustainable agriculture through improved nutrient retention, soil health, and climate resilience. By integrating biochar into ISFM practices, farmers can achieve higher productivity, reduce environmental impacts, and ensure long-term agricultural sustainability (**Table 2**).

**Table 2:** Effects of biochar on plant growth and crop productivity.

Plant Effect	Observed Biochar Benefit	Mechanism or Interaction
Nutrient Uptake	Enhanced uptake of N, P, K, and micronutrients	Improved nutrient availability and retention
Root Development	Better root penetration and architecture	Improved soil structure and reduced compaction
Stress Tolerance	Increased drought and salinity tolerance	Improved water retention and root-zone buffering capacity
Crop Yield	Increased yield in many cases (varies by crop and soil)	Nutrient synergy with organic fertilizers and enhanced soil health
Disease Resistance	Reduced incidence of soilborne pathogens	Microbial competition and pathogen suppression
Germination and Early Growth	Improved seedling establishment	Better moisture and nutrient availability

### 7.4 Challenges and limitations of biochar in practical agricultural use

Despite its wide-ranging benefits, the practical implementation of biochar in real-world agricultural systems faces several challenges. One of the primary concerns is the economic feasibility of large-scale biochar production and application. The cost of feedstock collection, pyrolysis equipment, labor, and transportation can make biochar unaffordable for smallholders and resource-limited farmers, especially in developing countries. Another limitation is the lack of standardization in biochar quality. The physical and chemical properties of biochar vary widely depending on feedstock type and pyrolysis conditions, making it difficult to predict its performance across different soils and crops. Without clear guidelines and quality benchmarks, farmers may face inconsistent outcomes.

In terms of scalability, while biochar can be produced from a variety of waste biomass, current production technologies may not be sufficient to meet the demand for broad-scale agricultural applications. Moreover, pyrolysis systems require technical knowledge, infrastructure, and energy inputs, which can limit adoption in remote or under-resourced areas. Environmental concerns also exist. While biochar is considered environmentally friendly, inappropriate feedstock sourcing or low-quality production methods could lead to the release of harmful pollutants during pyrolysis or result in biochar contaminated with heavy metals. Additionally, overapplication of biochar may alter soil pH or nutrient dynamics in ways that negatively affect crops.

Addressing these challenges requires a multi-pronged strategy, including the development of low-cost, decentralized biochar production systems, government incentives for sustainable waste utilization, and further research into optimal application rates and long-term impacts. Integrating biochar into sustainable agricultural systems must therefore balance agronomic potential with economic and ecological practicality.

## 8 Challenges and future directions

Despite the promising potential of biochar as a sustainable soil amendment, several key challenges and knowledge gaps remain that must be addressed to maximize its benefits. Research efforts should focus on the long-term impacts of biochar on specific crops, soil types, and agricultural systems. While short-term studies have demonstrated its positive effects on nutrient retention, soil health, and microbial activity, questions persist regarding the sustainability of these effects across multiple growing seasons. Evaluating whether biochar’s benefits persist or diminish over time, particularly in high-intensity farming systems with high nutrient demands, is crucial. Additionally, crop-specific responses to biochar, especially in staple crops such as maize, wheat, and rice, require further investigation to fine-tune application strategies for optimal productivity. Biochar’s performance varies significantly depending on soil characteristics, such as texture, pH, organic matter content, and nutrient profiles. For example, sandy soils, which are prone to nutrient leaching, may benefit substantially from biochar’s water-holding and nutrient retention capacity, while clayey soils may exhibit more limited improvements. Site-specific recommendations based on these soil properties are essential for tailoring biochar applications to different environments, particularly in degraded or marginal lands.

Another critical area for future research is the interaction of biochar with both organic and inorganic fertilizers. While biochar’s synergy with organic fertilizers like compost and manure has been well-documented, more studies are needed to understand its effects when used with synthetic fertilizers. Determining the optimal combinations and application rates to enhance nutrient use efficiency without disrupting soil chemistry or plant health is vital. Furthermore, the interaction of biochar with other soil amendments, such as mulches, cover crops, and green manures, presents additional opportunities to enhance soil health and resilience. The economic feasibility and scalability of biochar application are key considerations for its widespread adoption. Biochar production depends on the availability of feedstocks, pyrolysis technology, and transportation logistics. Developing efficient supply chains and regional production facilities that utilize locally available biomass can help reduce costs and improve scalability. Additionally, financial incentives, such as subsidies and carbon credits, can offset the high initial costs associated with biochar production and application, encouraging adoption among farmers. Research into the cost-benefit ratio of biochar under different cropping systems and soil conditions will provide valuable insights into its economic viability.

Scalable biochar application also depends on the development of mechanized tools for its uniform distribution in large fields. Manual application is labor-intensive and adapting existing agricultural machinery to handle biochar’s unique properties, such as its low density and fine particle size, is necessary for large-scale use. Policymakers and agricultural organizations can support scalability through education, demonstration projects, and policies that integrate biochar into sustainable farming and climate resilience frameworks. Another major consideration is the biochar’s role in addressing climate change. Its carbon sequence is well-documented, but more research is needed on its long-term behavior in different climatic and agricultural conditions. Understanding how biochar interacts with soil components over time, including potential issues such as heavy metal accumulation from certain feedstocks, is essential. Additionally, biochar’s potential to reduce greenhouse gas emissions, such as nitrous oxide and methane, under varying agricultural practices, including no-till farming and agroforestry, requires further investigation.

To maximize biochar’s agronomic and environmental benefits, optimizing its application involves determining the best feedstocks, pyrolysis conditions, and application rates for different soil types and crops. Feedstock selection is crucial, as it influences biochar’s chemical composition and stability. For example, wood-based biochar is ideal for carbon sequestration due to its high carbon content and structural stability, whereas manure-based biochar is more nutrient-rich, making it suitable for immediate soil fertility improvement. Pyrolysis conditions, particularly temperature, affect biochar properties such as porosity, surface area, and nutrient content. High-temperature pyrolysis produces biochar with high carbon stability, which is beneficial for long-term soil improvement, while low-temperature pyrolysis yields biochar with greater nutrient content and microbial stimulation potential. Determining the optimal pyrolysis conditions for different agricultural objectives is critical for balancing short-term soil fertility improvements with long-term carbon storage.

Application rates also need to be carefully calibrated to avoid unintended negative effects. Excessive biochar application can alter soil pH, disrupt nutrient balances, or create physical barriers to root growth. Conversely, under-application may not deliver the desired improvements. Site-specific research and field trials are necessary to establish appropriate application rates based on soil properties, organic matter content, and crop requirements. Biochar’s effectiveness is enhanced when integrated with other sustainable agricultural practices, such as composting, crop rotation, and reduced chemical fertilizer use. When combined with compost, biochar improves nutrient retention and microbial activity, leading to enhanced soil fertility and reduced greenhouse gas emissions during compost decomposition. Similarly, biochar complements crop rotation by supporting diverse microbial populations and improving soil structure, which facilitates nutrient cycling and root development.

Reducing chemical fertilizer use in conjunction with biochar applications can improve nutrient use efficiency while minimizing environmental pollution. Biochar absorbs and gradually releases nutrients, reducing leaching and volatilization losses. Its synergy with organic fertilizers further supports nutrient cycling and reduces dependence on synthetic inputs, making it a cornerstone of integrated soil fertility management (ISFM) strategies. Scaling up biochar production and application in large-scale agricultural systems requires addressing logistical, economic, and technological challenges. Establishing regional biochar production hubs that utilize locally available biomass can enhance supply chain efficiency. Developing mobile pyrolysis units and improving centralized biochar plants will help make biochar production more accessible, especially in rural areas. Economic incentives, such as subsidies, carbon credits, and inclusion in sustainable agriculture programs, can offset the high initial costs of biochar production and encourage adoption. Additionally, integrating biochar into existing farming practices, such as composting or nutrient management programs, can enhance its economic viability by reducing standalone costs.

Future research should also focus on the environmental sustainability of large-scale biochar production. Ensuring that biochar production uses renewable feedstocks and does not compete with food production or lead to deforestation is critical. Certification schemes and sustainability guidelines can help ensure that biochar production aligns with environmental and social goals.
